# Qualitative and quantitative characterization of the Rhesus monkey (*Macaca mulatta*) penis

**DOI:** 10.1590/S1677-5538.IBJU.2025.9906

**Published:** 2025-02-25

**Authors:** Marcelo Abidu-Figueiredo, Edilaine F. Alves, Carla B. M. Gallo, Waldemar S. Costa, Luciano A. Favorito, Francisco J. B. Sampaio

**Affiliations:** 1 Universidade do Estado do Rio de Janeiro Unidade de Pesquisa Urogenital Rio de Janeiro RJ Brasil Unidade de Pesquisa Urogenital, Universidade do Estado do Rio de Janeiro – UERJ, Rio de Janeiro, RJ, Brasil;; 2 Universidade Federal Rural do Rio de Janeiro Departamento de Anatomia Animal e Humana Seropédica RJ Brasil Departamento de Anatomia Animal e Humana, Universidade Federal Rural do Rio de Janeiro - UFRRJ, Seropédica, RJ, Brasil

**Keywords:** Penis, Macaca mulatta, Models, Theoretical, anatomy and histology [Subheading]

## Abstract

**Background:**

Knowledge of the anatomy of laboratory animals is important for experimental research. Erectile dysfunction has been studied using the penises of different laboratory animals such as rats, mice, rabbits, dogs, etc. However, these animals have penises with different characteristics to the human penis. If these differences are not taken into account, the conclusions may be questionable. The Rhesus monkey (*Macaca mulatta*), due to its similarities to humans, could be a good model.

**Objective:**

To characterize and quantify the components of the penis of the Rhesus monkey (*Macaca mulatta*), qualifying it as a model for experimental studies.

**Methods:**

Ten adult Rhesus monkey penises were fixed in 10% buffered formalin and processed for paraffin embedding. Histological sections 5-μm thick were made and stained using histochemical techniques. We assessed the thickness of the tunica albuginea, and in the erectile tissue, the following parameters were analyzed: in the corpus cavernosum (CC): total area, area densities of collagen fibers, muscle fibers and elastic system fibers; in the corpus spongiosum (CS): area densities of collagen fibers, muscle fibers and elastic system fibers. Histomorphometric analyses were carried out on photomicrographs by using ImageJ software.

**Results:**

The penis of the Rhesus monkey (*Macaca mulatta*) has a single CC. The tunica albuginea was thicker in the dorsal region (1.11 ± 0.03 mm) than in the ventral region (0.87 ± 0.01 mm). The quantitative analysis of the CC showed the following values: total area (20.33 ± 5.67 mm²), collagen fibers (24.00 ± 4.00%), muscle fibers (31.52 ± 9.93%) and elastic system fibers (8.46 ± 3.20%). The quantitative analysis of the CS showed the following values: collagen fibers (52.50 ± 11.76%), muscle fibers (10.50 ± 6.36%) and elastic system fibers (15.07 ± 4.78%).

**Conclusion:**

The predominance of muscle tissue over connective tissue in the corpus cavernosum, similar to what is observed in humans, qualifies the Rhesus monkey penis as a good experimental model for erectile dysfunction.

## INTRODUCTION

Different studies classify the penis into two types according to different species: fibroelastic, in which the penis increases in length and changes little in diameter; and muscle-cavernous, in which the penis increases in both length and diameter (1, 2).

The human penis is muscle-cavernous and composed of the glans, two corpora cavernosa (CC) and the corpus spongiosum (CS). The CCs are completely surrounded by the tunica albuginea, which is composed of collagen fibers and fibers from the elastic system. The elastic fibers usually form an irregular network on which the collagen fibers rest. The CCs contain smooth muscle fibers and fibrous connective tissue that form the wall of the sinusoids. The CS surrounds the urethra and, similar to the CC, is composed of smooth muscle and connective tissue fibers. Smooth muscle is an essential component of the sinusoids in the CC, CS and glans penis (3-5).

Rats and mice are widely used as experimental models for studies on the urogenital system (6-8). However, the rat penis is of the fibroelastic type, as well as has a bone component and a predominance of connective tissue in the CC (1, 5, 9).

Among non-human primates, the Rhesus monkey (*Macaca mulatta*) is the species most used in scientific research. This is due to the similarities between the Rhesus monkey and the human being (10). The penis of the Rhesus monkey is of the muscle-cavernous type (10).

There are several works in the literature on the structure of the Rhesus monkey's organs: lower urinary tract (11), kidneys (12-14), prostate (15), vas deferens (16), immune (17) and lymphatic systems (18), bone tissue (19) and metabolic disorders (20).

A detailed description of the Rhesus monkey penis, as well as a characterization of the different structures that compose it, has not yet been done. A study along these lines, showing very similar parameters to the human penis, could justify the use of the Rhesus monkey penis as an experimental model.

This study aims to determine, using qualitative and quantitative methods, the morphological and histological characteristics of the penis of the Rhesus monkey (*Macaca mulatta*), qualifying it as a model for experimental studies in humans.

## MATERIAL AND METHODS

The study protocol was approved by the Animal Ethics Committee (CEUA Protocol No. 014/2015) of the Institute of Biological and Health Sciences of the Federal Rural University of Rio de Janeiro (UFRRJ).

Ten penises from adult Rhesus monkeys were collected, fixed in 4% buffered formalin and processed for paraffin embedding. Histological sections 5-μm thick were obtained from each sample and stained using the following histochemical techniques: hematoxylin and eosin to evaluate tissue integrity; Picrosirius red without polarized light to analyze the density of collagen fiber areas in the CC and CS; Picrosirius red with polarized light for qualitative analysis of collagen fibers in the tunica albuginea; Masson's trichrome for the thickness of the tunica albuginea, analysis of the area of the CC and the area density of the muscle fibers in the CC and CS; and Weigert's resorcin-fuchsin with previous oxidation for analysis of the area density of the fibers of the elastic system in the CC, CS and qualitative analysis in the tunica albuginea.

All histomorphometric analyses were carried out using ImageJ^®^ software, version 1.50i, loaded with its own plug-in (http://www.imagej.nih.gov/ij).

Histomorphometric analyses of the thickness of the tunica albuginea and the area of the CC of the penis, including the tunica albuginea, were carried out using X12 magnification photomicrographs taken with a stereomicroscope (SteREO Discovery.V8, Zeiss, Göttingen, Germany) coupled to a digital camera (Axiocam 506 color, Carl Zeiss, Göttingen, Germany).

The "straight line" tool was used to analyze the thickness of the tunica albuginea of the CC (expressed in mm), in which three random linear measurements were taken in the dorsal and ventral regions, and the average of the measurements was taken to obtain the thickness of the tunica albuginea in each region. The "freehand" tool was used to analyze the area of the CC (expressed in mm²), in which three measurements were taken in each field, and these were averaged to obtain the area of the CC. For both analyses, five sections of each sample were analyzed, and one field of each section was observed, for a total of five fields in each sample.

Histomorphometric analyses of the area densities of collagen fibers, muscle fibers and elastic system fibers (expressed as percentages) in the CC and CS were carried out using photomicrographs at X600 magnification, obtained by using a microscope (Olympus BX51, Tokyo, Japan) equipped with a digital camera (Olympus DP71, Tokyo, Japan). The area density of these parameters was estimated using the quantification evaluation method, by superimposing a 100-point test grid (multipurpose test system) on the magnified images on the video monitor screen (21). For all these analyses, five sections of each sample were analyzed and five random fields of each section were observed, for a total of 25 fields in each sample.

## RESULTS

**Figure 1:**
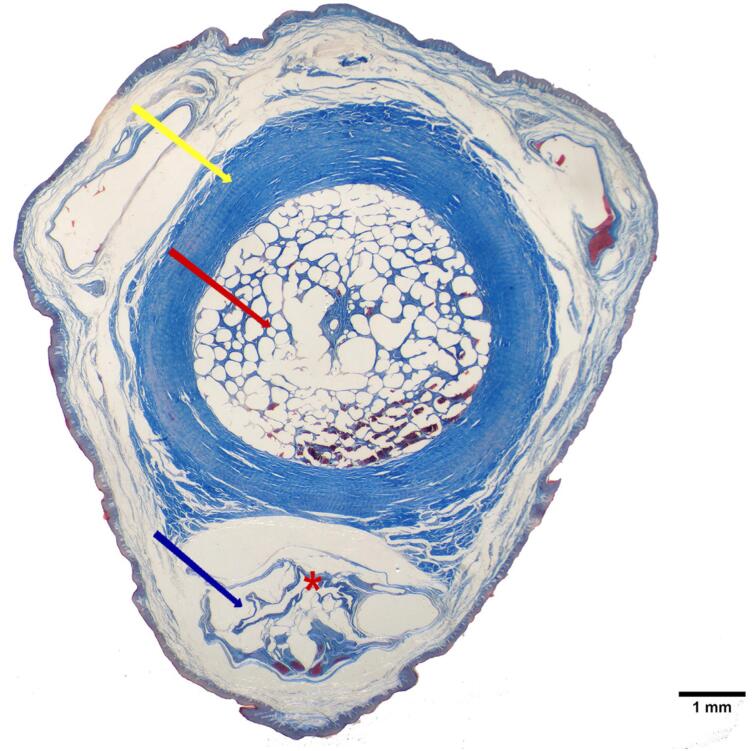
Photomicrograph of the Rhesus monkey penis. Tunica albuginea (yellow arrow), corpus cavernosum (red arrow), corpus spongiosum (black arrow), urethra (asterisk). Masson's Trichrome, X12.

**Figure 2:**
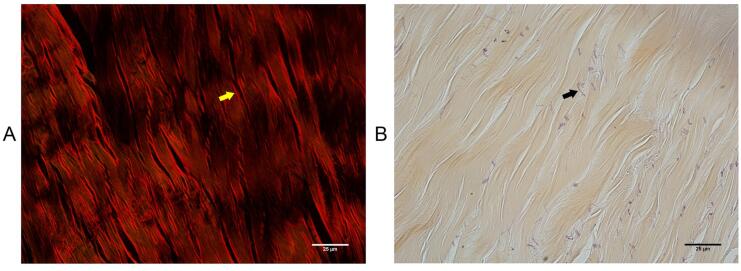
Photomicrographs of the tunica albuginea of the Rhesus monkey penis. A) Thick collagen fibers (yellow arrow), type-I collagen, picrosirius red with polarization, X600. B) Elastic system fibers are less numerous (black arrow). Weigert's resorcin-fuchsin, X600.

**Figure 3:**
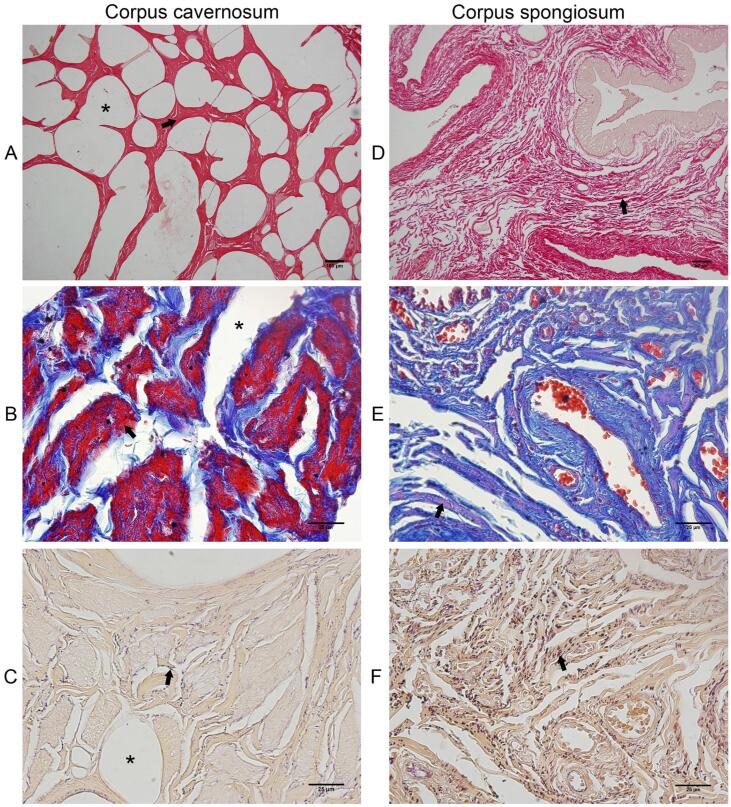
Photomicrographs of the corpus cavernosum and corpus spongiosum of the Rhesus monkey penis. A) Corpus cavernosum, sinusoids (*), collagen fibers (arrow), picrosirius red, X100. B) Smooth muscle fibers (arrow), sinusoids (*), Masson's trichrome, X600. C) Elastic system fibers (arrow), sinusoids (*), Weigert's resorcin-fuchsin, X600. D) Corpus spongiosum, collagen fibers (arrow), picrosirius red, X200. E) Smooth muscle fibers (arrow), Masson's trichrome, X600. F) Elastic system fibers (arrow), Weigert's resorcin-fuchsin, X600.

In the qualitative analysis, we observed a single CC (Figure-1), surrounded by a thick tunica albuginea, composed mainly by type-I collagen, due to the presence of thick collagen fibers with red birefringence and less numerous elastic system fibers (Figure-2). The tunica albuginea was thicker in the dorsal region (+21.62%) than in the ventral region (Table-1). The analysis of the density of smooth muscle, collagen and elastic fibers areas, the Corpus Cavernosum showed a predominance of smooth muscle fibers (49%) over collagen (38%) and elastic system fibers (13%). On the other hand, the Corpus Spongiosum showed a predominance of collagen (67%), over elastic system fibers (19%) and smooth muscle fibers (14%). The quantitative analyses of the components of the CC and CS are shown in Table-1. Figure-3 shows the different components of CC and CS.

**Table 1:** Data on the parameters analyzed in the corpus cavernosum and corpus spongiosum of the penis of the Rhesus monkey (Macaca mulatta)

Data	Values (mean ± standard deviation)	
	Corpus cavernosum parameters	Corpus spongiosum parameters
**Thickness of the dorsal tunica albuginea (mm)**	1.11 ± 0.03	-
**Thickness of the ventral tunica albuginea (mm)**	0.87 ± 0.01	-
**Total area (mm²)**	20.33 ± 5.67	-
**Sv [collagen fibers] (%)**	24.00 ± 4.00	52.50 ± 11.76
**Sv [muscle fibers] (%)**	31.52 ± 9.93	10.50 ± 6.36
**Sv [elastic system fibers] (%)**	8.46 ± 3.20	15.07 ± 4.78

Sv = surface densities

## DISCUSSION

Knowledge of the anatomy of laboratory animals is essential for experimental research, allowing the animal to be adapted to the study being carried out. Erectile dysfunction, for example, has been studied using the penises of different species (22), including rats and mice (23, 24), rabbits (25) and monkeys (26).

Although these species have several characteristics similar to those of the human penis, there are differences in the penile anatomy of these animals compared to the human penis (1, 5, 9).

The rat's CC is predominantly composed of connective tissue (1, 5, 9). In contrast, the male penis is predominantly muscular. This could make it difficult to make a comparison using these animals as a model. As in rats, the penises of dogs and cats also have a bony component in their cranial part, which is considered to be an ossified part of the CC and is part of the erectile components (27, 28).

The human penis has two paired corpora cavernosa (4), as is the case with other species such as rats, dogs and rabbits, in which the penis has two distinct corpora cavernosa, partially separated by the median septum (27). The penis of the Rhesus monkey was not clearly divided into two corpora cavernosa. De Siqueira et al. (29) also observed a single non-septated CC in the marmoset. Despite being unique, the components of the Rhesus monkeys CC are very similar to those of humans, such as the predominant presence of smooth muscle fibers in relation to the fibrous component. This characteristic allows for a more reliable comparison. The proportion of smooth muscle fibers in the CC of the human penis is approximately 40% (30). Similarly, the CC of the Rhesus monkey penis had 49% more muscle fibers than the other components analyzed.

The tunica albuginea of the CC in humans is a structure composed of inner circular and outer longitudinal layers which have bundles of thick collagen fibers resting on a network of irregularly arranged elastic fibers (3, 31, 32). In the CC of the penis of the Rhesus monkey, we observed a thick tunica albuginea enveloping the entire CC, which showed thick collagen fibers birefringent in red when observed under polarized light, which may characterize type-I collagen fibers. The analysis of the tunica albuginea in adult men showed that the dorsal region was thicker than the ventral region, making the ventral region a more vulnerable area, as in humans (33). Even from the human fetal period, the tunica albuginea already shows a morphological difference in relation to the dorsal and ventral regions. Gallo et al. (34) studied human fetuses between 13- and 36-weeks post-conception and reported that the thickness of the tunica albuginea was greater in the dorsal than ventral region. In our study, the tunica albuginea of the Rhesus monkey penis also showed this characteristic of being thicker dorsally than ventrally (+21.62%).

It is known that the elastic system fibers form an interconnected network in order to keep the collagen fiber bundles together (32). The density of elastic system fibers in the CS of the Rhesus monkey penis was higher than in the CC (+ 43.86%). These characteristics are very similar to those described in the human penis, where there is also a predominance of elastic fibers in the CS (31).

All the similarities between the different structures when comparing the penis of the Rhesus monkey and humans, fully justify the use of this animal as a good experimental model for the study of the human penis.

## CONCLUSIONS

The predominance of smooth muscle fibers over connective tissue in the corpus cavernosum, similar to what is observed in humans, qualifies the penis of the Rhesus monkey as a good experimental model.
